# Case Report: Paraneoplastic Hashimoto's Encephalopathy Associated With Lymphomatosis Cerebri With Periodic Synchronous Discharges Resembling Creutzfeldt–Jakob Disease

**DOI:** 10.3389/fneur.2021.701178

**Published:** 2021-08-10

**Authors:** Ryota Amano, Setsuro Tsukada, Shota Kosuge, Satoshi Yano, Kenjiro Ono, Makoto Yoneda, Katsumi Taki

**Affiliations:** ^1^Department of Internal Medicine, Fujiyoshida Municipal Medical Center, Fujiyoshida, Japan; ^2^Division of Neurology, Department of Internal Medicine, Showa University School of Medicine, Tokyo, Japan; ^3^Faculty of Nursing and Social Welfare Science, Fukui Prefectural University, Fukui, Japan

**Keywords:** Hashimoto's encephalopathy, lymphomatosis cerebri, anti-NH2-terminal of α-enolase antibody, periodic synchronous discharge, Creutzfeldt–Jakob disease

## Abstract

Hashimoto's encephalopathy (HE) is an autoimmune encephalopathy that presents with various clinical symptoms, including cognitive deterioration, convulsive seizures, and personality changes. HE is associated with thyroid autoimmunity; however, few cases have been reported to develop as paraneoplastic syndrome. Herein, we report the case of a 73-year-old woman with onset of rapidly progressive dementia. Brain magnetic resonance imaging showed diffuse T2 hyperintensity areas involving the bilateral cerebral white matter, right midbrain tegmental area, left cerebral peduncle, and right middle cerebellar peduncle without clear diffusion hyperintensities and gadolinium enhancement. Her neurological symptoms worsened rapidly, and she presented with the apallic syndrome. Electroencephalogram showed periodic synchronous discharge, suggestive of Creutzfeldt–Jakob disease. However, a brain biopsy revealed infiltration of atypical lymphoid cells expressing CD20, and the anti-NH2 terminal of the α-enolase antibody was detected, diagnosing the complication with lymphomatosis cerebri and HE. High-dose intravenous methylprednisolone therapy and oral prednisolone with whole cranial irradiation enabled her to have simple conversations and consume food orally; however, severe cognitive impairment persisted. Although HE is a rare complication of malignant lymphoma, clinicians should be aware that it could be strongly suspected if the clinical symptoms worsen in the absence of imaging changes.

## Introduction

Hashimoto's encephalopathy (HE), a steroid-responsive disorder, is an autoimmune encephalopathy associated with Hashimoto's thyroiditis in the euthyroid state (1, 2). As HE presents a variety of clinical symptoms, clinicians sometimes misdiagnose it as other neurological diseases, such as seizures, Alzheimer's disease, limbic encephalitis, psychiatric diseases, or Creutzfeldt–Jakob disease (CJD) (3–6). At present, elevation of serum anti-thyroid autoantibodies, such as the anti-thyroid peroxidase (TPO) antibody and/or anti-thyroglobulin (Tg) antibody, is useful and essential for the diagnosis of HE; however, the anti-TPO antibody or anti-Tg antibody is known to be detected in approximately 10% of normal adults (7–9). The specificity of serum diagnosis of HE by anti-thyroid autoantibodies is low. However, Yoneda et al. reported that the serum anti-NH2 terminal of the α-enolase (NAE) antibody is a specific biomarker for HE (with specificity of 91% and sensitivity of 50%) (10, 11). Therefore, serum diagnosis of HE has recently become easier.

Lymphomatosis cerebri (LC), a rare variant of primary central nervous system lymphoma (PCNSL) that represents 2–3% of all brain tumors (12), was initially described in 1999 (13). Only less than 50 LC cases have been reported by 2019 (14). PCNSL is generally easy to diagnose with the mass formation in the brain with homogeneous contrast effects on gadolinium-enhanced MRI (15). In contrast, diagnosing LC is challenging as it shows diffuse T2 high-intensity signals without obvious contrast effect or mass formation even if a contrast effect is present (16).

As HE sometimes shows diffuse non-specific T2 high-intensity signals in the bilateral cerebral white matter on brain MRI (3), it is often impossible to discriminate between HE and LC using only image findings. However, HE is an autoimmune disease, whereas LC is a malignant neoplastic disease; therefore, HE and LC are completely different diseases, and to the best of our knowledge, LC-related HE has not been reported before. Herein, we report a case of paraneoplastic encephalopathy with anti-NAE antibody complicated with LC, which was diagnosed using brain biopsy. The clinical presentation was similar to that of CJD and responded to steroid therapy. We believe that, similar to this case, HE develops as a paraneoplastic neurological syndrome of LC.

## Case Presentation

A 73-year-old woman with a history of type 2 diabetes and non-tuberculous mycobacterial infection presented to our hospital with subacute progressive dementia characterized by nausea, dizziness, headaches, loss of recent memory, and behavioral changes for 3 months. On admission, her vital signs were within the normal range. Neurological examinations confirmed disturbance of consciousness [Glasgow Coma Scale (GCS) score of 14 (E4V4M6)], increased deep tendon reflex in the left upper limb and bilateral lower limbs, positive pathological reflexes (Babinski and Chaddock reflexes), cerebellar ataxia in the right upper limb, apathy, perseveration, acalculia, and finger agnosia. We did not observe cranial nerve palsies, muscle weakness, or sensory disturbances.

**Figure 1:**
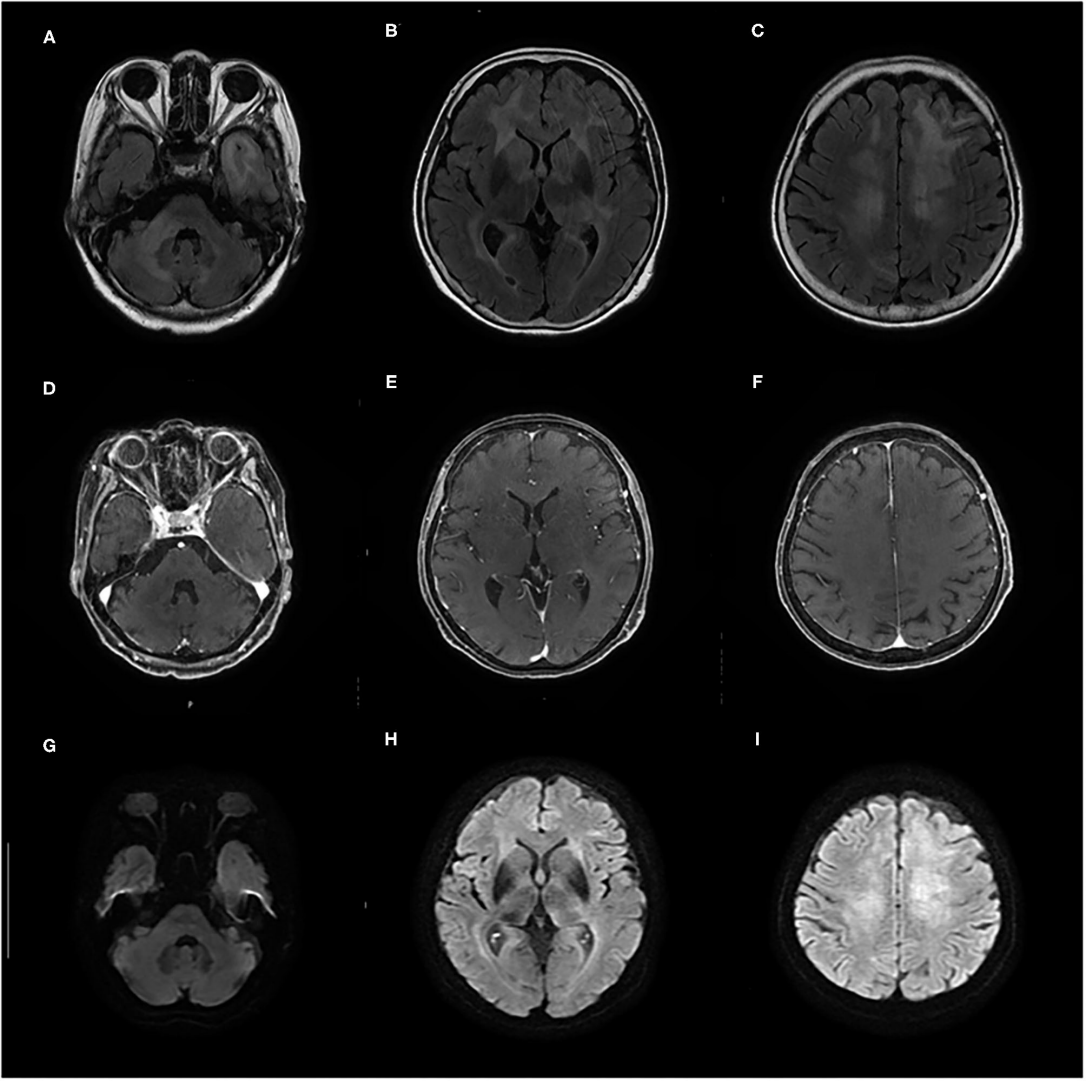
**(A–F)** Contrast-enhanced brain MRI taken on admission shows diffuse T2/fluid-attenuated inversion recovery (FLAIR) hyperintense lesions in bilateral cerebral white matter, left temporal pole, and right middle cerebellar peduncle without gadolinium enhancement. **(G–I)** On diffusion-weighted imaging (DWI), these lesions show faintly high intensities that can be explained by T2 shine-through.

Initial laboratory tests did not reveal any specific abnormalities. Complete blood count, liver function, and renal function were within the reference range (RR). Thyroid function was in a euthyroid state, and no elevation of serum anti-TPO antibody and anti-Tg antibody was observed in electrochemiluminescence immunoassay (<9.0 and <10.8 IU/ml, respectively; RR <16.0 and <28.0 IU/ml, respectively). The concentrations of lactate dehydrogenase (LDH) and soluble interleukin-2 receptor (sIL-2R) were not elevated (134 IU/L and 206 U/ml, respectively; RR = 100–220 IU/L and 121–613 U/ml, respectively), and human immunodeficiency virus was negative. Cerebrospinal fluid (CSF) analyses showed elevated total protein levels (TP = 52 mg/dl, RR = 10–40 mg/dl) and slightly high levels of sIL-2R (61 U/ml, RR <60.4 U/ml); however, the LDH concentration was 42 U/ml (RR = 8–50 U/ml). CSF analyses also demonstrated normal cell counts (1 leucocyte/μl) without atypia and no amplification of polymerase chain reaction for the John Cunningham (JC) virus. Brain MRI on initial presentation revealed diffuse non-enhancing T2/fluid-attenuated inversion recovery hyperintense lesions in the bilateral cerebral white matter, left temporal pole, and right middle cerebellar peduncle (Figures 1A–F). On diffusion-weighted imaging (DWI), these lesions showed faintly high intensities; however, they could be explained by T2 shine-through (Figures 1G–I).

**Figure 2:**
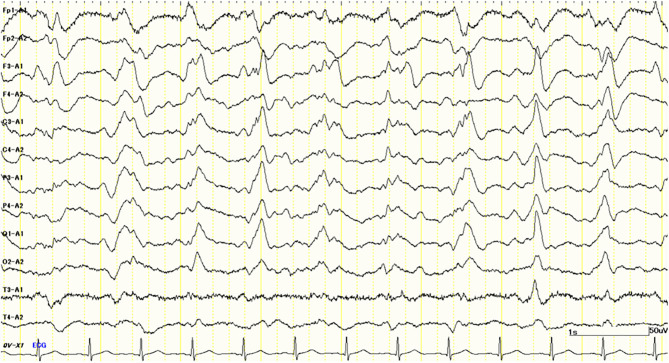
Electroencephalography (EEG) study 1.5 months after admission shows periodic synchronous discharge (PSD) suggesting Creutzfeldt–Jakob disease (CJD).

Her cognitive impairments progressively worsened 1 month after admission. She simultaneously presented with a GCS score of E1M1V4 and the apallic syndrome. Despite our best efforts, we could not obtain any specific findings to confirm the diagnosis. One and a half months after admission, her electroencephalogram (EEG) showed bilateral periodic synchronous discharge (PSD), typically suggestive of CJD (Figure 2). To confirm the diagnosis as “CJD,” we measured the CSF total tau protein and 14-3-3 protein levels. However, no elevation of the concentrations of these proteins in the CSF was observed (total tau = 708 pg/ml, RR <1,300 pg/ml; 14-3-3 protein <500 μg/ml, RR <500 μg/ml). Only a half-day after initiating high-dose methylprednisolone (mPSL) therapy (1,000 mg/day), her consciousness improved rapidly; therefore, we treated her with an additional 2 days of high-dose mPSL therapy. She maintained a good state of consciousness during the 3 days of therapy. However, her consciousness worsened soon after high-dose mPSL therapy. These clinical characteristics suggest a lower possibility of CJD.

**Figure 3:**
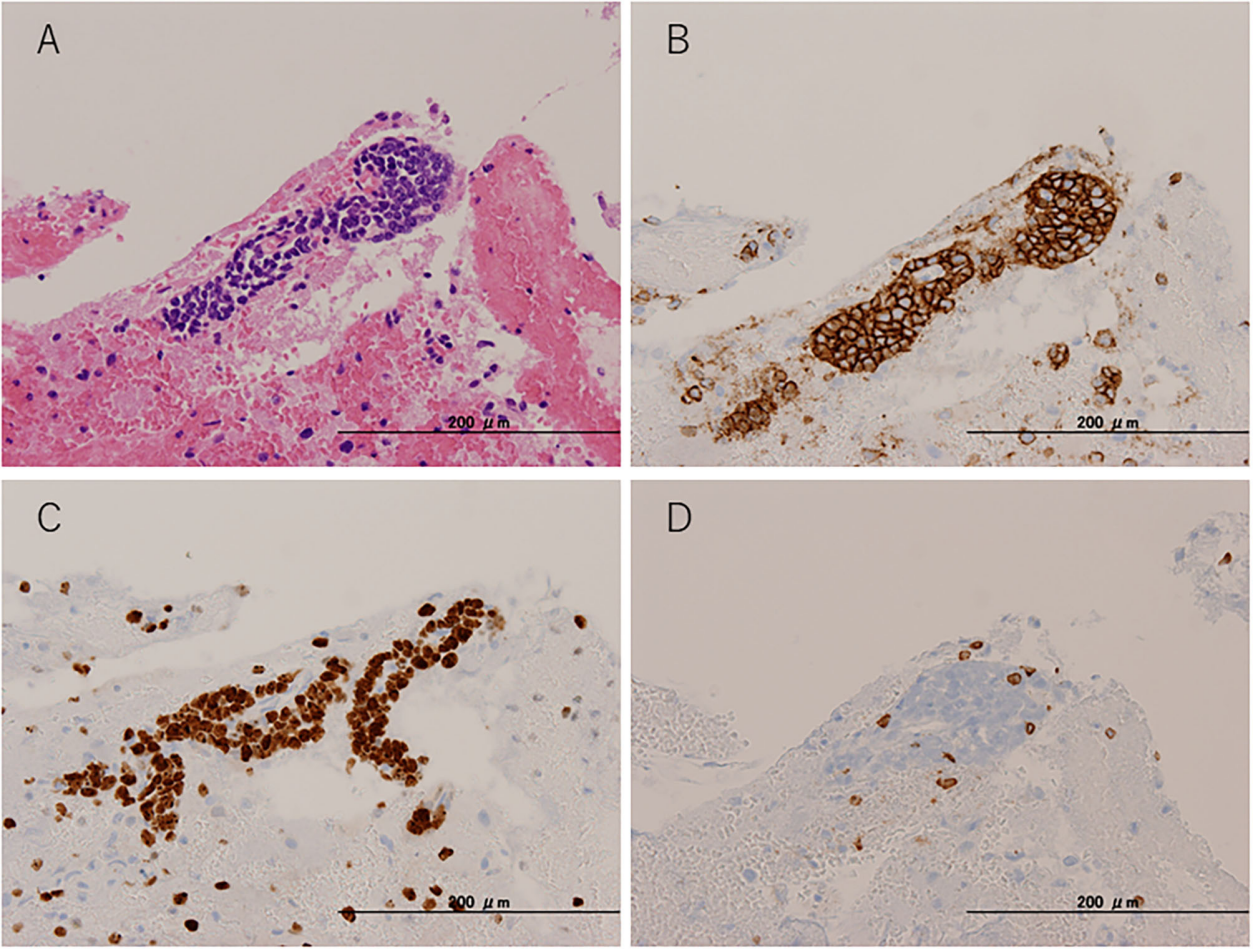
Pathological findings. Brain biopsy from the left frontal lobe. **(A)** Hematoxylin and eosin staining. **(B)** CD20 immunohistochemical staining. **(C)** Ki-67 immunohistochemical staining. **(D)** CD3 immunohistochemical staining.

To pathologically evaluate the abnormalities seen on MRI, we performed a brain biopsy of the left frontal lobe. Pathological findings revealed infiltration of atypical lymphoid cells with large and irregularly shaped nuclei (Figure 3A). These atypical lymphoid cells were positive for cluster differentiation (CD) 20, with 80–90% of the Ki-67 proliferation index (Figures 3B,C). A few CD3-positive reactive T cells were also observed and did not show irregularities (Figure 3D). Moreover, the CSF data were reexamined when the brain biopsy showed clearer abnormalities (cell count = 10 leucocytes/μl, TP = 68 mg/dl, sIL-2R = 121 U/ml, LDH = 66 U/ml) than before. Based on these pathological findings, CSF abnormalities, and the distribution of white matter lesions on MRI, we diagnosed her with primary central nervous system B cell lymphoma of the LC type.

**Figure 4:**
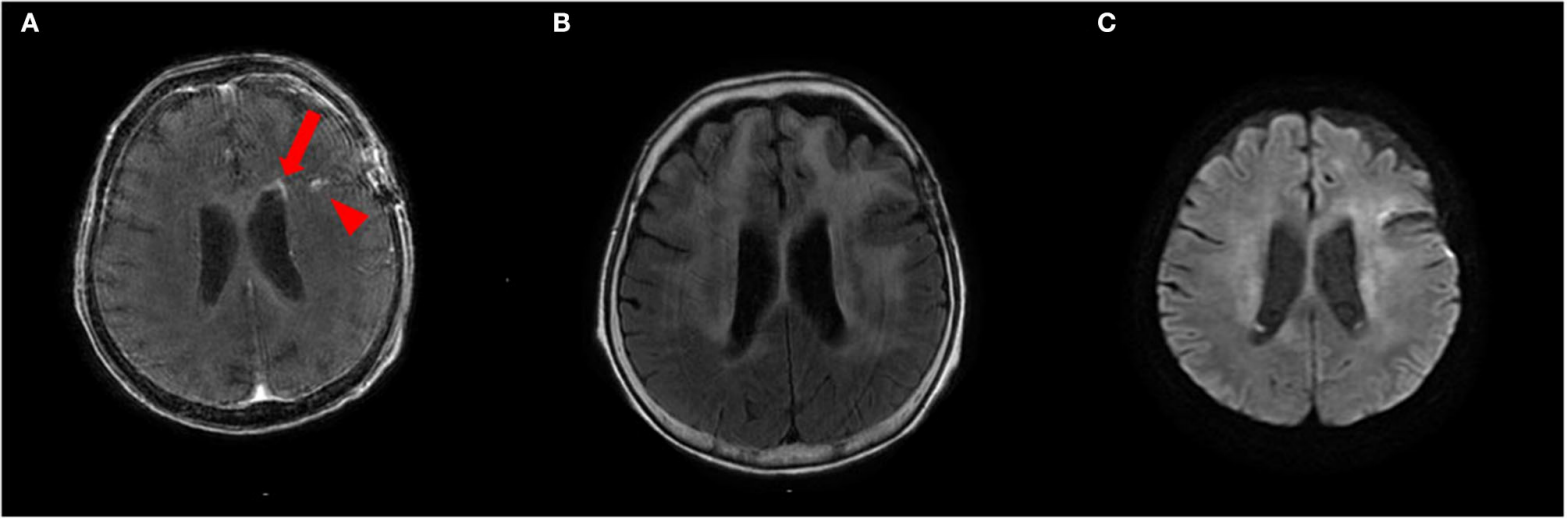
**(A,B)** Contrast-enhanced brain MRI recorded 1 month after brain biopsy shows a spotty gadolinium enhancement in the left periventricular white matter (*arrow*) and post-biopsy scar (*arrowhead*) **(A)** with T2 hyperintensities **(B)**. **(C)** This lesion does not show abnormal hyperintensities on diffusion-weighted imaging (DWI).

To treat LC, we administered two courses of high-dose mPSL (1,000 mg/day for 3 days), followed by 60 mg (2 mg/kg) prednisolone (PSL) for 63 days with a taper of every 5 mg for 7 days and whole cranial irradiation. Contrast-enhanced MRI performed 1 month after brain biopsy revealed a spotty gadolinium enhancement in the left periventricular white matter without high intensity on DWI (Figures 4A–C). Two months after the treatment initiation, the anti-NAE antibody was detected in the serum before mPSL treatment, revealing the presence of HE. We performed thyroid sonography, and it was characterized by isoechogenicity, very slightly internal heterogeneity, no diffuse goiter, and a few cysts (Supplementary Figure 1). Moreover, a second check showed the serum anti-TPO antibody and anti-Tg antibody to be within the RR in chemiluminescent immunoassay (0.72 and <0.50 IU/ml, respectively; RR <4.11 and <5.61 IU/ml, respectively). At that time, she recovered from the apallic syndrome, had simple conversations, and consumed food orally. However, her consciousness worsened again when PSL was reduced to 15 mg/day. No evident new abnormality, including aggravation of LC, was found on the re-performed contrast-enhanced MRI. After another high-dose mPSL therapy, her consciousness improved; therefore, we considered the re-exacerbation of her consciousness as due to HE relapse, even though we performed a third check of the serum anti-TPO and anti-Tg antibodies, which were within the RR in chemiluminescent immunoassay (0.44 and <0.50 IU/ml, respectively). Although her consciousness improved, severe cognitive impairment persisted, and she still needed careful assistance for all her daily living activities. The clinical timeline is shown in Supplementary Figure 2.

## Discussion

We present a case of a rare complication of the anti-NAE antibody-related autoimmune encephalopathy and LC showing a CJD-like clinical presentation and PSD on EEG. Since the detection of the anti-NAE antibody and the presence of PSD on EEG suggested the same mechanisms of encephalopathy as HE, we diagnosed this anti-NAE antibody-related autoimmune encephalopathy as that.

EEG is occasionally used in neurological disorders associated with convulsive seizures and impaired consciousness. PSD on EEG can be observed particularly in diseases such as CJD, subacute sclerosing panencephalitis (SSPE), and Alzheimer's disease (17, 18). The EEG of HE shows a variety of abnormal findings in ~90% of cases, and the basal waves tend to slow activities. However, some HE cases present PSD on EEG and require differentiation from CJD, similar to our case (6, 19, 20).

As LC has been recently reported as a new disease concept, a variant type of PCNSL, as reported by Bakshi et al., it is often overlooked as another disease (13). As far as we investigated in PubMed using the term “lymphomatosis cerebri,” only 71 cases in 43 articles were reported until 2020, and no complications with HE were found (13, 14, 16, 21–60).

In these reports, seven mentioned the use of EEG for LC (13, 27, 32, 48). Most of these cases have reported generalized diffuse slowing activities with non-specific abnormalities (13, 27, 32). Deutsch et al. reported that four cases of LC on EEG were observed only with diffuse slowing without PSD, and they described that the presence of PSD could be an important differential point between LC and CJD (27). However, Revero et al. reported a case showing PSD in the “T” cell type of LC (48). In the present case, although the possibility of the B cell type of LC-derived PSD could not be ruled out, it was appropriate that PSD was derived from the anti-NAE antibody-related autoimmune encephalopathy diagnosed as HE.

At present, it is said that the positive serum levels of the anti-thyroid antibodies are essential for HE diagnosis (61). In our case, although repetitive measurements of the anti-thyroid autoantibodies in different ways were performed three times, all of these were negative. Admittedly, the presence of Hashimoto's thyroiditis could not be denied from the slight heterogeneity based on the thyroid ultrasonographic findings, but, to our knowledge, no report has made the diagnosis of HE only from the ultrasonographic findings without positive anti-thyroid autoantibodies. Moreover, unless the paraneoplastic syndrome exists, the neoplasm and autoimmune encephalopathy rarely co-occur. Therefore, the possibility of the thyroid autoimmunity causing the autoimmune encephalopathy was very low. Strictly speaking, the term HE might not be used for this case. At present, paraneoplastic encephalopathy with anti-NAE antibody may be more appropriate. However, the presence of anti-NAE antibodies has high specificity (~90%) for the serum diagnosis of HE (10, 11). Hence, because of the detection of the anti-NAE antibody and PSD on EEG, the same mechanisms of autoimmune encephalopathy were suspected as the anti-thyroid autoantibody-positive “normal” HE. Although anti-thyroid autoantibodies were not detected, the detection of anti-NAE antibodies permitted the diagnosis of HE or some autoimmune encephalopathy extremely similar to HE, and continued steroid treatment led to the improvement of clinical symptoms.

In our case, the possibility that the primary intracerebral inflammation by HE caused secondary oncogenesis of lymphocytes was low. If HE caused malignant lymphoma, the anti-thyroid autoantibodies should have been detected similar to normal HEs. Patients with malignant lymphomas have a potential risk of various autoimmune diseases (62, 63). Furthermore, the clinical presentation of malignant lymphoma sometimes develops from complicated autoimmune disorders before the tumor itself (64). Although the mechanisms of anti-NAE antibody production are not clear, our case might have developed as a paraneoplastic neurological syndrome of LC.

The elevation of sIL-2R levels in PCNSL is well-known. For diffuse large B cell lymphoma of PCNSL, Sasagawa et al. reported that the cutoff value of sIL-2R in the CSF was 60.4 U/ml (sensitivity = 94.7%, specificity = 84.6%) (65). It might not have been typical that the concentrations of initial sIL-2R in the CSF in our case were only slightly high; although, its elevation may be observed non-specifically in neurosarcoidosis and meningitis (66). Currently, the levels of IL-10 in the CSF have been reported as a more useful biomarker for the initial screening of PCNSL (cutoff = 3 pg/ml, sensitivity = 94.7%, specificity = 100%) (65). If the levels of IL-10 in the CSF were measured during the first screening, LC could have been diagnosed earlier.

## Conclusion

In conclusion, we report a case of a rare complication of HE and LC presenting with CJD-like clinical presentation and PSD. Although the initial presentation of subacute progressive dementia and EEG features was consistent with CJD, the CSF abnormalities, particularly total tau protein and 14-3-3 protein, and steroid responsiveness were not typical of CJD. Physicians should be aware of the possibility of PCNSLs and assess the total tau protein, 14-3-3 protein, sIL-2R, and IL-10 in the CSF at the first screening. Moreover, when the clinical presentation worsens without the aggravation of image findings, physicians should consider the complications of HE. Accurate and early diagnosis and appropriate treatment can improve the clinical outcomes.

## Data Availability Statement

The original contributions presented in the study are included in the article/Supplementary Material, further inquiries can be directed to the corresponding author/s.

## Ethics Statement

The studies involving human participants were reviewed and approved by Fujiyoshida Municipal Medical Center. The patient provided their written informed consent to participate in this study. Written informed consent was obtained from the individual's next of kin for the publication of any potentially identifiable images or data included in this article.

## Author Contributions

RA, ST, SK, SY, and KO were the attending physicians of the patient, collected the patient data, and decided on a treatment policy. MY measured the anti-NAE antibody levels. KT assessed thyroidal function and proposed critical opinion regarding thyroid disease. RA and KT wrote the first version of this manuscript and played major roles in the conception of the manuscript. All authors have read and approved the final version of the manuscript.

## Conflict of Interest

The authors declare that the research was conducted in the absence of any commercial or financial relationships that could be construed as a potential conflict of interest.

## Publisher's Note

All claims expressed in this article are solely those of the authors and do not necessarily represent those of their affiliated organizations, or those of the publisher, the editors and the reviewers. Any product that may be evaluated in this article, or claim that may be made by its manufacturer, is not guaranteed or endorsed by the publisher.

## Supplementary Material

Supplementary Figure 1 B-mode of ultrasonographic image of the thyroid gland.
