# Supplementary material for: Case Report: Paraneoplastic Hashimoto's Encephalopathy Associated With Lymphomatosis Cerebri With Periodic Synchronous Discharges Resembling Creutzfeldt–Jakob Disease
Source: Front Neurol. 2021 Aug 10;12:701178. doi: 10.3389/fneur.2021.701178 (PMC8384121; doi:10.3389/fneur.2021.701178)
Supplement: Supplementary file 1 [file Data_Sheet_1.PDF]

CARE case report guidelines

CARE Checklist of information to include when writing a case report

(cc) BY-NC-ND

Topic	Item	Checklist item description	Reported on Line
Title	1	The diagnosis or intervention of primary focus followed by the words "case report"	Page1, Title
Key Words	2	2 to 5 key words that identify diagnoses or interventions in this case report, including "case report"	Page1, line 14~15
Abstract (no references)	3a	Introduction: What is unique about this case and what does it add to the scientific literature?	Abstract, line 31~33
	3b	Main symptoms and/or important clinical findings	Abstract, line 20~28
	3c	The main diagnoses, therapeutic interventions, and outcomes	Abstract, line 28~31
	3d	Conclusion—What is the main "take-away" lesson(s) from this case?	Abstract, line 31~33
Introduction	4	One or two paragraphs summarizing why this case is unique (**may include references**)	Introduction, line 57~61
Patient Information	5a	De-identified patient specific information.	Case presentation, line 64
	5b	Primary concerns and symptoms of the patient.	Case presentation, line 65.66
	5c	Medical, family, and psycho-social history including relevant genetic information	Case presentation, line 64
	5d	Relevant past interventions with outcomes	N/A
Clinical Findings	6	Describe significant physical examination (PE) and important clinical findings.	Case presentation, line 68~72
Timeline	7	Historical and current information from this episode of care organized as a timeline	Supplementaly figure 1
Diagnostic Assessment	8a	Diagnostic testing (such as PE, laboratory testing, imaging, surveys).	Case presentation, paragraph 4,5
	8b	Diagnostic challenges (such as access to testing, financial, or cultural)	N/A
	8c	Diagnosis (including other diagnoses considered)	Case presentation, paragraph 4.5
	8d	Prognosis (such as staging in oncology) where applicable	N/A
Therapeutic Intervention	9a	Types of therapeutic intervention (such as pharmacologic, surgical, preventive, self-care)	Case presentation, paragraph 5
	9b	Administration of therapeutic intervention (such as dosage, strength, duration)	Case presentation, paragraph 5
	9c	Changes in therapeutic intervention (with rationale)	Case presentation, paragraph 5
Follow-up and Outcomes	10a	Clinician and patient-assessed outcomes (if available)	Case presentation, paragraph 5
	10b	Important follow-up diagnostic and other test results	Case presentation, paragraph 5
	10c	Intervention adherence and tolerability (How was this assessed?)	N/A
	10d	Adverse and unanticipated events	N/A
Discussion	11a	A scientific discussion of the strengths AND limitations associated with this case report	N/A
	11b	Discussion of the relevant medical literature **with references**.	Discussion, paragraph 1~6
	11c	The scientific rationale for any conclusions (including assessment of possible causes)	Discussion, paragraph 4,5
	11d	The primary "take-away" lessons of this case report (without references) in a one paragraph conclusion	Conclusion, line 168~172
Patient Perspective	12	The patient should share their perspective in one to two paragraphs on the treatment(s) they received	N/A
Informed Consent	13	Did the patient give informed consent? Please provide if requested	Yes ☑ No ☐
