# Supplementary figures and images for: Case Report: Paraneoplastic Hashimoto's Encephalopathy Associated With Lymphomatosis Cerebri With Periodic Synchronous Discharges Resembling Creutzfeldt–Jakob Disease
Source: Front Neurol. 2021 Aug 10;12:701178. doi: 10.3389/fneur.2021.701178 (PMC8384121; doi:10.3389/fneur.2021.701178)

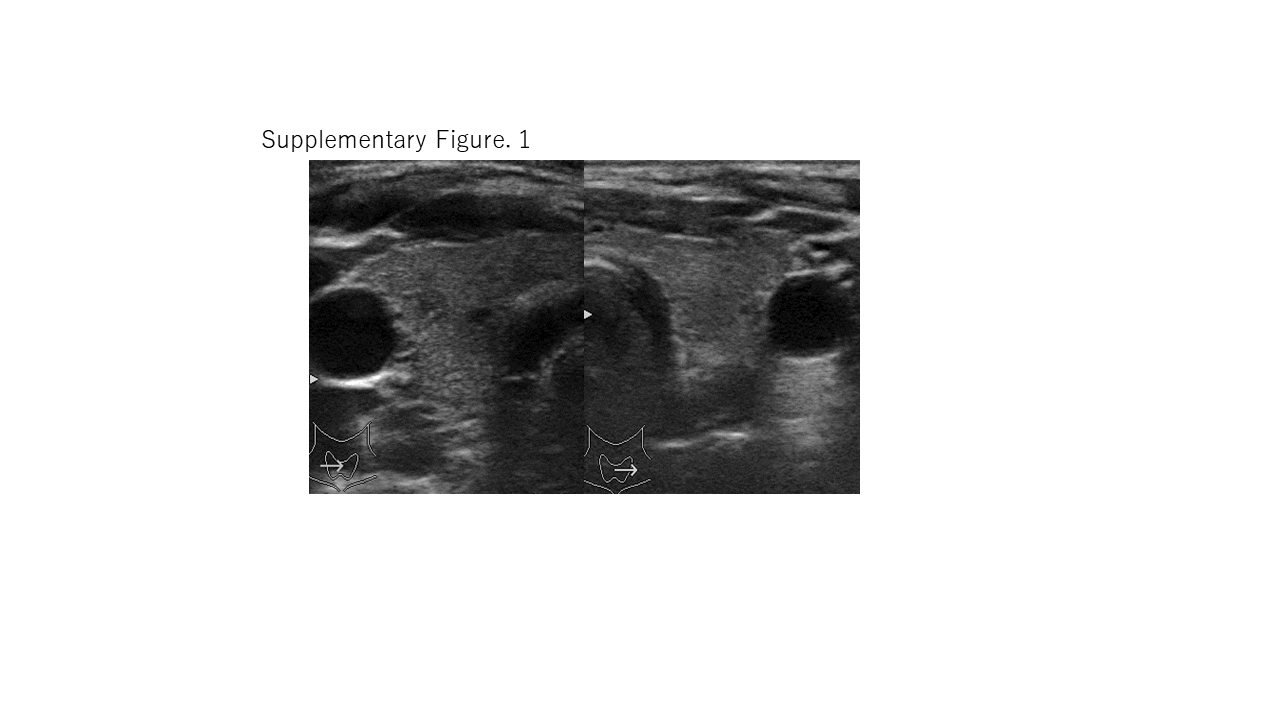

Supplement: Supplementary Figure 1 — B-mode of ultrasonographic image of the thyroid gland. [file Image_1.TIF]

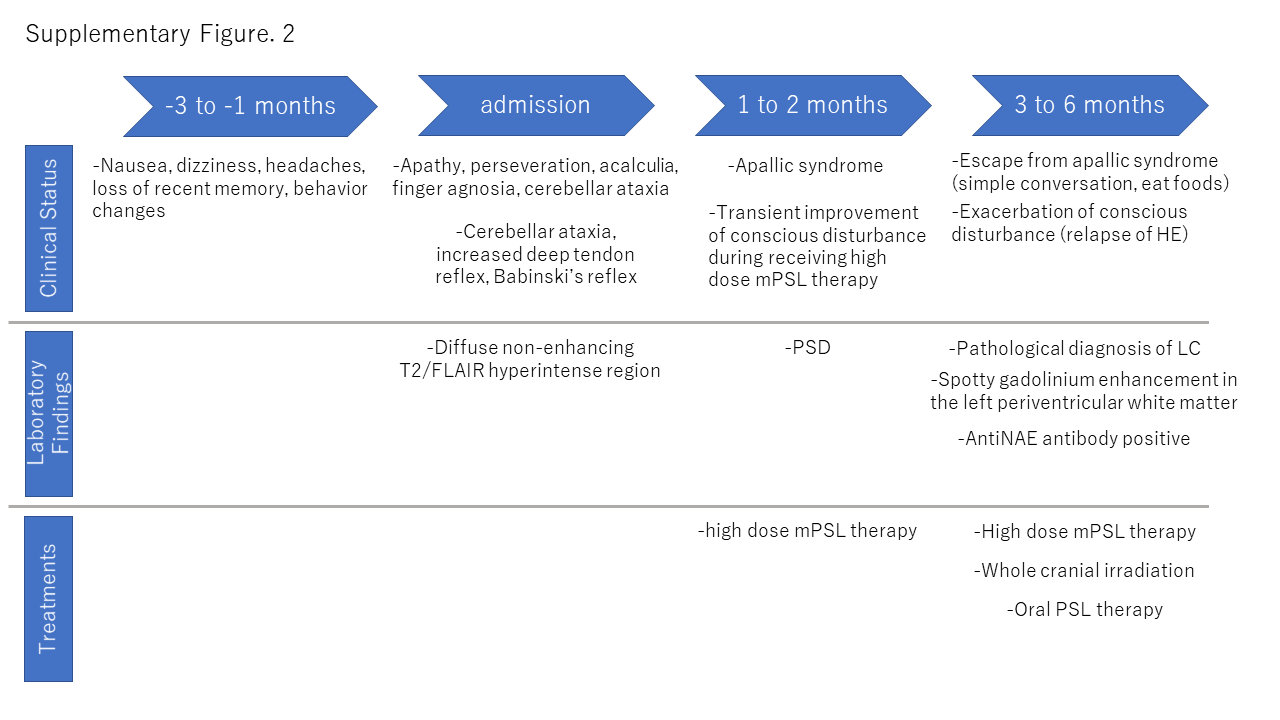

Supplement: Supplementary file 3 [file Image_2.TIF]
